# L1CAM as a prognostic marker in stage I endometrial cancer: a validation study

**DOI:** 10.1186/s12885-016-2631-4

**Published:** 2016-08-04

**Authors:** Elisabeth Smogeli, Ben Davidson, Milada Cvancarova, Arild Holth, Betina Katz, Bjørn Risberg, Gunnar Kristensen, Kristina Lindemann

**Affiliations:** 1 Department of Gynecologic Oncology, Norwegian Radium Hospital, Oslo University Hospital, PB 4953 Nydalen 0424, Oslo, Norway; 2 Department of Pathology, Oslo University Hospital, Norwegian Radium Hospital, Oslo, Norway; 3 Institute of Clinical Medicine, University of Oslo, Faculty of Medicine, Oslo, Norway; 4 Faculty of Health Sciences, Oslo and Akershus University College of Applied Sciences, Oslo, Norway; 5 Institute for Cancer Genetics and Informatics, Norwegian Radium Hospital, Oslo University Hospital, Oslo, Norway; 6 Department of Medical Oncology, Crown Princess Mary Cancer Care Center, Westmead Hospital, Westmead, NSW 2145 Australia; 7 NHMRC Clinical Trials Centre, University of Sydney, Locked Bag 77, Camperdown, NSW 1450 Australia

**Keywords:** L1CAM, Endometrial cancer, Prognostic marker

## Abstract

**Background:**

L1 cell adhesion molecule (L1CAM) overexpression has been reported to be strongly associated with poor prognosis in early stage endometrial cancer (EC).

We aimed at the validation of L1CAM as a marker of poor prognosis in an independent study population.

**Methods:**

Patients with endometrioid EC FIGO stage I, were treated at Oslo University Hospital between 2005 and 2012. L1CAM expression was detected by immunohistochemistry with >10 % L1CAM staining defined as positive. Risks of relapse and death were estimated as hazard ratios (HRs) with 95 % confidence intervals (95 % CI).

**Results:**

Of 450 patients, 388 (86 %) were evaluable for L1CAM expression and 35 (9 %) were L1CAM positive. After follow-up for a median time of 4.8 years (0.1–8.8), 33 (8 %) patients had recurred. 6/35 (17 %) L1CAM positive patients relapsed compared to 27/353 (8 %) L1CAM-negative patients. There were 7 (20 %) deaths in the L1CAM positive group, and 34 (10 %) in the negative group. In multivariate analysis, controlled for age and FIGO stage, L1CAM positivity was not significantly associated with the risk of relapse (HR 2.08, 95 % CI: 0.85–5.10, *p =* 0.11) or death of all-cause (HR 1.81, 95 % CI: 0.79–4.11, *p =* 0.16). In patients who were not treated with chemotherapy, L1CAM was significantly associated with risk of relapse (HR 2.9; 95 % CI: 1.08–7.56; *p =* 0.04).

**Conclusion:**

Our report confirms that L1CAM is associated with a more aggressive tumortype and more distant relapses. The overall recurrence rate in this population was low as were the absolute differences between L1CAM positive and negative patients. In this independent study sample, L1CAM failed to be a clinically relevant marker of poor prognosis in stage I endometrioid endometrial carcinoma.

## Background

Endometrial cancer (EC) is the most common malignancy in the female genital tract in the Western world, and the fourth most common cancer in women after breast, lung and colorectal cancer. Two different clinicopathological subtypes of EC are recognized, the estrogen-related (type I) endometrioid adenocarcinoma, and the non-estrogen related (type II) non-endometrioid, mostly serous adenocarcinoma [1]. Endometrioid adenocarcinomas represent 80 % of endometrial carcinomas and patients are often diagnosed at an early stage with disease localized to the uterus [1]. These patients will have a favourable prognosis with five-year overall survival rates of up to 85 %. Despite the overall good prognosis some patients will eventually relapse and may ultimately die of the disease. The most important prognostic factors in stage I disease are age, grade of differentiation, myometrial invasion, lymphovascular space invasion (LVSI) and histological type. Traditionally these risk factors have been used to categorize patients into risk groups and to tailor adjuvant treatment. The current clinical challenge is to identify patients at high risk for distant relapse as they have a substantially worse prognosis.

Recently, L1 cell adhesion molecule (L1CAM) has been suggested as a biomarker that may discriminate a subset of highly aggressive tumors with adverse clinical outcome [2, 3]. L1CAM belongs to the immunoglobulin (Ig) supergene family and is a transmembrane glycoprotein of 200–220 kDA. This cell adhesion molecule plays an important role in nervous system development, including neuronal migration, and differentiation. In endometrial cancer, L1CAM staining has been described as diffuse and localized in tumor cells adjacent to the stroma, suggestive of the role of L1CAM in the migration and invasion of tumor cells [4]. Retrospective studies have shown that L1CAM expression in even small areas of endometrioid adenocarcinomas is associated with adverse outcome [2, 3, 5]. Zeimet et al. included stage I endometrioid endometrial carcinoma only and reported 17.7 % L1CAM positive tumors [3]. L1CAM positivity was associated with increased risk of relapse, especially distant relapse, and risk of death. More recently, these findings were confirmed in an analysis of L1CAM expression in endometrial cancers from two randomised controlled trials (PORTEC-1 and -2) [2] and in a multicentre retrospective study conducted by the ENITEC consortium [6]. However, the prevalence of 7 % positive tumors was substantially lower compared to the initial report.

The aim of this study was to analyse L1CAM expression in an independent series of stage I endometrioid endometrial carcinoma and to study the association of L1CAM expression with risk of relapse and death.

## Methods

### Patients and follow-up

Patients were selected from a validated quality assurance database at the Department of Gynecological Cancer at the Oslo University Hospital. The database covers all patients treated at Oslo University Hospital (The Norwegian Radium Hospital, Ullevål University Hospital and Rikshospitalet) for endometrial cancer. The database is linked to Statistics Norway and individual survival data are available through this linkage. The database provides detailed information on the primary diagnosis, the preoperative work-up, comorbidity, surgical treatment, adjuvant treatment, incident relapse, localization of relapse, and date of death. We included all patients with endometrioid endometrial cancer stage I, treated at the Oslo University Hospital between 2005 and 2012. Histopathological diagnosis was confirmed by review of the hematoxylin-eosin slides by three surgical pathologists (BD, BR, BK) specialized in gynecologic pathology at the Norwegian Radium Hospital. Patients with synchronous ovarian cancer were excluded (*n =* 33). For this study the optimal slide from each individual case was selected, based on the presence of sufficient amount of tumor, good fixation and the presence of normal myometrium as a control.

Patients were staged according to the 2009 FIGO classification and categorized as low (stage IA, grade 1 and 2), intermediate (stage IA grade 3, or stage IB grade 1 and 2), or high risk (stage IB grade 3). They were treated with surgery and adjuvant chemotherapy according to the national treatment policy. In general, low risk patients were treated with extrafascial hysterectomy and bilateral salpingo-oophorectomy alone, while intermediate and high-risk patients underwent lymph node staging. After surgery, high-risk patient were offered systemic treatment with platinum-based chemotherapy. Patients were followed every 3 months for the first 2 years and every 6 months for the next 3 years. Visits included thorough clinical examination and vaginal ultrasound, supplemented by CT or MR scan on clinical indication.

### Immunohistochemical staining and evaluation of expression

The formalin fixed paraffin-embedded (FFPE) tissue blocks were collected and cut into 3-4μm sections and mounted on Superfrost slides. The sections were analyzed for L1CAM protein expression using the Dako FLEX+ protocol (Dako, Glostrup, Denmark). The L1CAM antibody was a mouse monoclonal antibody (clone 14.10, cat. # SIG-3911) from Covance (Princeton NJ), applied at 1:300 dilutions. Antigen retrieval was performed in low pH buffer (Dako). Visualization was achieved using 3′3-diaminobenzidine tetrahydrochloride substrate (DAB) and hematoxylin counterstaining. Positive control consisted of a high-grade serous carcinoma shown to be positive in antibody testing, and was satisfactory in all reactions. Negative controls consisted of slides stained with IgG1k murine melanoma immunoglobulin (Sigma-Aldrich, St. Louis MO; cat. # M9035) at the same concentration.

**Fig. 1:**
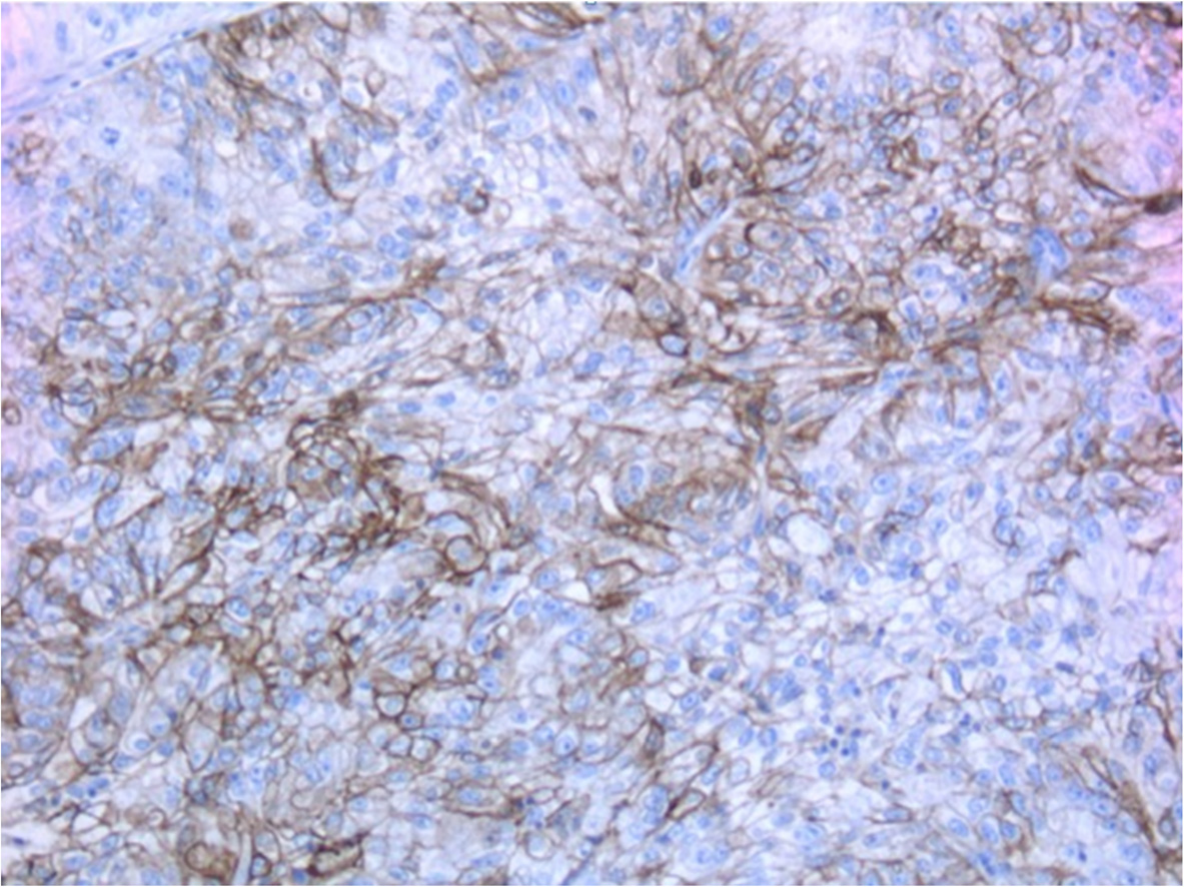
Immunohistochemical staining of L1CAM positive tumor

Staining extent was scored as positive (>10 % of cells) (Fig. 1) vs. negative (≤10 % of cells). This cut-off has previously been reported to result in the strongest model and was confirmed by Bosse et al. [2, 3]. Scoring was performed by one gynecologic pathologist (BD), which was blinded for clinical outcome.

### Statistical analysis

This study is a ”prospective-retrospective” design that used archived tumor specimen as suggested by Simon et al. [4]. Our power calculation was based on results reported in the literature. Relapse rates of 3 % in L1CAM negative tumors and 50 % in L1CAM positive tumors have been reported [3]. Given a statistical power of 80 % and alpha of 5 % we would have needed 17 patients in each group and 10 relapses in total. Further, an absolute difference of 37 % in the number of deaths has been reported previously [3], with 40 % death rate in the L1CAM positive group and 3 % death rate in the negative group. With the same statistical assumptions as above (HR = 3), we would have needed 24 patients in each group and 10 deaths to replicate the results. Therefore, given the sample size available and the above stated assumptions, our study was sufficiently powered.

Continuous variables were descried as median and range. Categorical variables were presented with counts and proportions. Crude associations between L1CAM negative and L1CAM positive patients and categorical variables were assessed with χ^2^ test.

When studying the risk of relapse, follow-up time was calculated from the date of EC diagnosis until date of relapse, date of death from any cause or end of follow-up, August 31, 2014, whichever occurred first.

For risk of death, follow-up time was calculated from the date of EC diagnosis until date of death from any cause or end of follow-up, whichever occurred first. Survival curves were plotted with the Kaplan-Meier method. The log rank test was used to compare survival between the groups. Crude hazard ratios (HRs) of relapse and death with 95 % confidence intervals (CI) associated with L1CAM overexpression were calculated using Cox proportional hazard models. The proportionality assumption was tested using Schoenfeld’s residuals. All variables revealing prognostic significance in the univariate analysis or previously have been reported to be associated with risk of relapse or death, were included in the multivariable model. The final multivariate model was adjusted for FIGO stage and age < vs ≥ 60 years. An alternative model was adjusted for age as attained age at the diagnosis of endometrial cancer and contained L1CAM status, FIGO stage and grade. However the estimates remained unchanged. In exploratory subgroup analysis by treatment with adjuvant chemotherapy or not, we studied the association of L1CAM expression with the risk of relapse. P-values <0.05 were considered statistically significant and all tests were two-sided.

The analyses were performed using IBM SPSS version 22 (SPSS, Chicago, IL) and the STATA statistical package, version 11.0, (Stata Corp LP, Texas, USA).

## Results

### Patient and characteristics and treatment

**Fig. 2:**
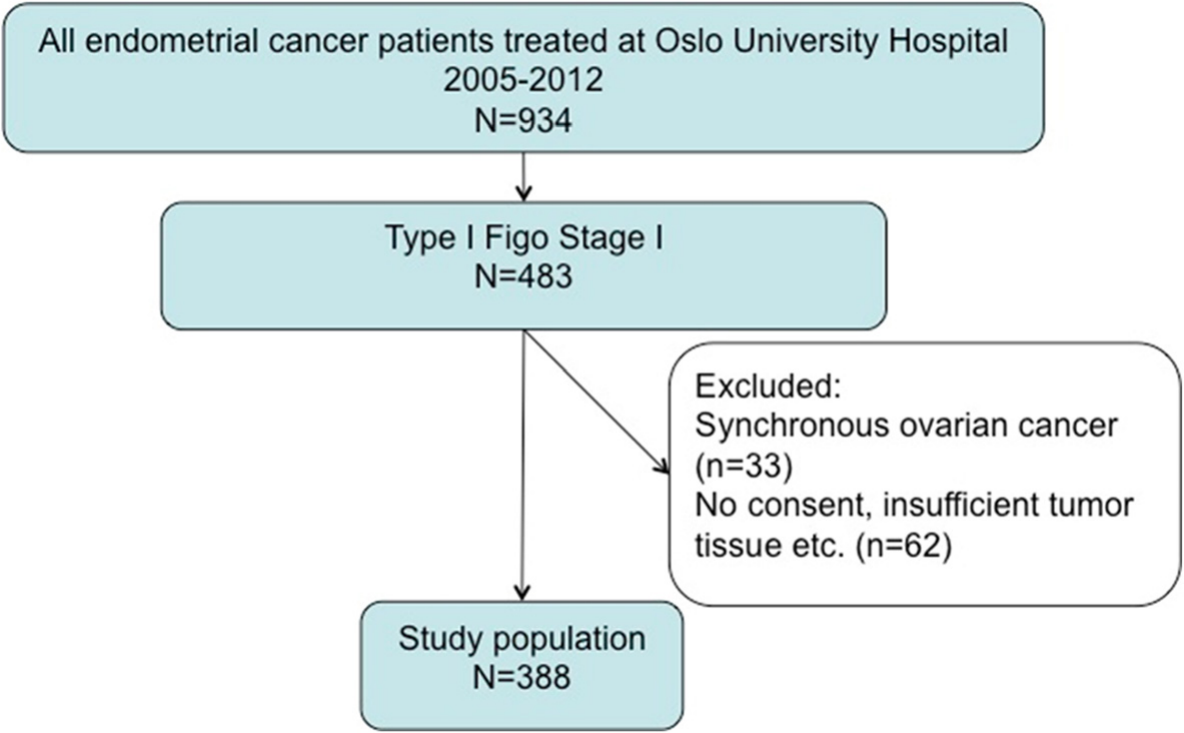
Study population

During our study period, 450 patients underwent surgery for FIGO stage I endometrioid endometrial adenocarcinomas. In 62 patients (14 %) the amount of tumor tissue archived was insufficient, the material had undergone autolysis or we did not obtain informed consent. In total, 388 (86 %) were evaluable for the L1CAM expression (Fig. 2). The median age of the study population at diagnosis was 66.8 years (range 39–91 years) and median follow-up time was 4.8 years (range 0.1–8.8). Baseline characteristics of the study population are displayed in Table 1. Of the total cohort, 186 (48 %) patients had undergone pelvic lymphadenectomy and of these 107 (28 %) also had para-aortic lymphadenectomy. The majority of patients, 350 (90 %) received no adjuvant treatment, while 38 (10 %) patients received adjuvant chemotherapy and one patient underwent postoperative pelvic radiotherapy.

**Table 1:** Baseline characteristics according to L1CAM status (*n =* 388)

Baseline characteristics	Total	%	L1CAM positive *n =* 35	%	L1CAM negative *n =* 353	%	*P*-value
Age at diagnosis							0.116
< 60	111	28,6	6	17,1	105	29,7	
> 60	277	71,4	29	82,9	248	70,3	
FIGO stage							0.223
1A	268	69,1	21	60,0	247	70,0	
1B	120	30,9	14	40,0	106	30,0	
Risk Groups							<0.001
Low	238	61,3	14	40,0	224	63,5	
Intermediate	120	30,9	13	37,1	107	30,3	
High	27	7,0	8	22,9	19	5,4	
Grading							<0.001
Grade I	231	59,5	9	25,7	222	62,9	
Grade II	103	26,5	11	31,4	92	26,1	
Grade III	54	13,9	15	42,9	39	11,0	
LVSI	371		35		336		0.756
Yes	61	16,4	7	20,0	54	16,1	
No	268	72,2	25	71,4	243	72,3	
Unknown	42	11,3	3	8,6	39	11,6	
Pelvic lymphadenectomy							0.016
No	202	52,1	12	34,3	190	53,8	
Yes	186	47,9	23	65,7	163	46,2	
Para-aortic lymphadenectomy							0.082
No	281	72,4	20	57,1	261	73,9	
Yes	107	27,6	15	42,9	92	26,1	
Adjuvant chemotherapy			35		353		<0.001
No	350	90,2	23	65,7	327	92,6	
Yes	38	9,8	12	34,3	26	7,4	
Diabetes			35		353		0.102
No	325	83,8	30	85,7	295	83,6	
Yes	61	15,7	4	11,4	57	16,1	
Unknown	2	0,5	1	2,9	1	0,3	
Obesity	375		34		341		0.127
BMI < 30	242	64,5	26	76,5	216	63,3	
BMI > 30	133	35,5	8	23,5	125	36,7	
Smoking	386		35		351		0.858
No	253	65,5	24	68,6	228	65,0	
Yes	56	14,5	4	11,4	52	14,8	
Unknown	77	19,9	7	20,0	70	19,9	

### L1CAM expression

Of the 388 cases available for immunohistochemical staining, 35 (9 %) stained positive for L1CAM, and 353 (91 %) were L1CAM negative. In 5 cases L1CAM expression was observed in 5–10 % of the cells. These were not considered as positive cases in the analysis.

L1CAM positivity was strongly associated with histological differentiation (*p <* 0.001), with an increasing proportion of L1CAM positive tumors with poorer differentiation. L1CAM positivity was also strongly associated with increasing risk grouping (*p <* 0.001). A significantly higher proportion of L1CAM positive patients had undergone pelvic lymphadenectomy (23/35 = 66 % vs 163/353 = 46 %, *p =* 0.027) or had received adjuvant chemotherapy (12/35 = 34 % vs 26/353 = 7 %, *p <* 0.001). L1CAM positive patients were slightly older compared to L1CAM negative patients, with a median age of 70.6 years (range 47.8–88.8) and 66.5 years (range 38.8–91.2), respectively, however, this difference was not statistically significant. There were no significant associations between L1CAM expression and FIGO stage, lymphovascular space invasion (LVSI), diabetes, smoking, or obesity as defined by BMI ≥30 kg/m^2^.

### Relapse and death

**Fig. 3:**
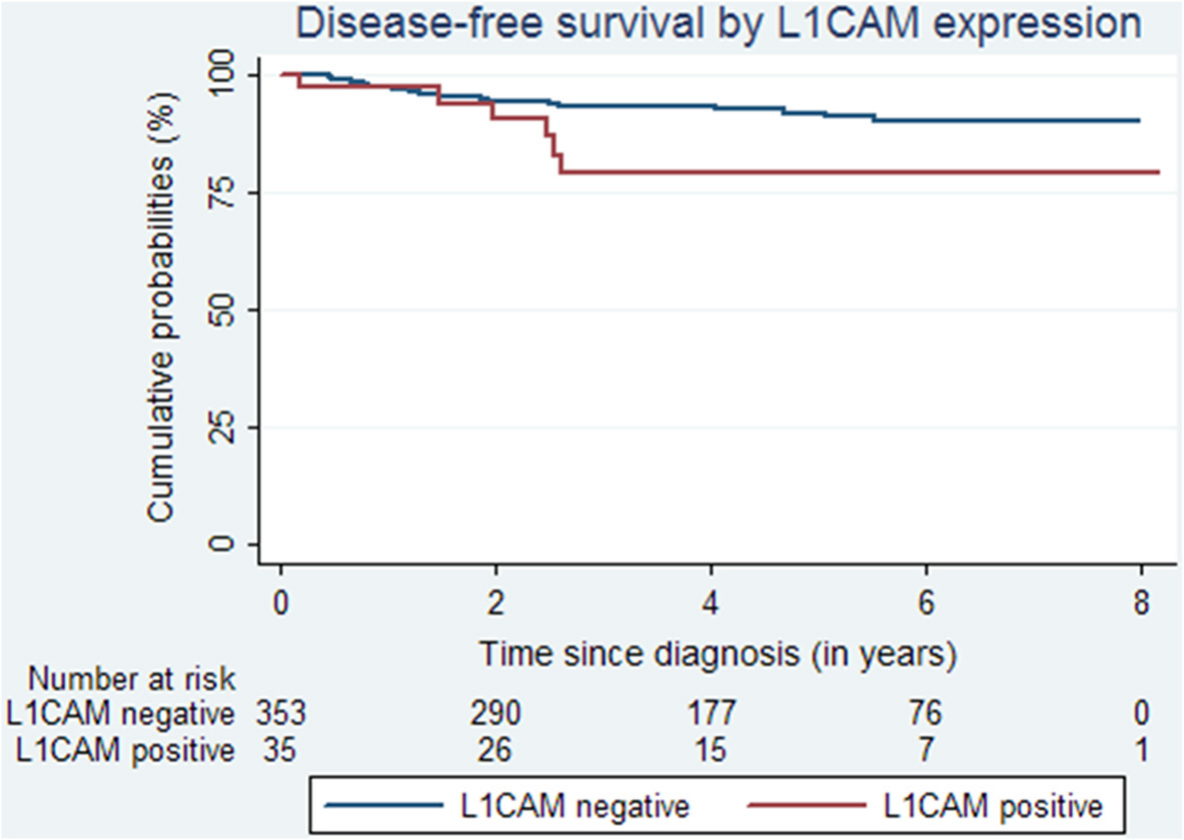
Disease-free survival by L1CAM expression

A total of 33 patients (8.5 %) recurred during follow-up (Table 2) and 29 of those had not received adjuvant chemotherapy. In the L1CAM positive group, half the relapses were isolated vaginal relapses and half were distant metastasis. In the L1CAM negative group, most relapses were isolated vaginal relapses and only 1 % recurred with distant metastasis. Median disease-free survival was not reached (Fig. 3). In subgroup analysis for adjuvant chemotherapy, 24 out of 327 L1CAM negative patients relapsed after no adjuvant chemotherapy compared to 5 out of 23 L1CAM positive patients. The distribution of relapses in those patients was similar to the cohort as a whole with more distant relapses in the L1CAM positive group (Table 3).

**Table 2:** Clinical outcome according to L1CAM expression (*n =* 388)

Event	L1CAM positive *n =* 35 (%)	L1CAM negative *n =* 353 (%)	Total *n =* 388 (%)
Relapse	6 (17)	27 (8)	33 (9)
Isolated vagina	3 (9)	18 (5)	21 (5)
Pelvic	0	4 (1)	4 (1)
Distant	3 (9)	5 (1)	8 (2)
Death	7 (20)	34 (10)	41 (11)

**Table 3:** Clinical outcome according to L1CAM expression in patients who did not receive adjuvant chemotherapy (*n =* 350)

Event	L1CAM positive *n =* 23 (%)	L1CAM negative *n =* 327 (%)	Total *n =* 350 (%)
Relapse	5 (23)	24 (7)	29 (8)
Isolated vagina	1 (4)	9 (9)	10 (3)
Pelvic	1 (4)	4 (1)	5 (1)
Distant	3 (13)	11 (3)	14 (4)
Death	6 (26)	3 (10)	38 (11)

In univariate analysis on the total group of patients, L1CAM positivity was not significantly associated with disease free survival (HR: 2.38, 95 % CI 0.98–5.77, *p =* 0.06), neither were FIGO stage nor age. In multivariate analysis, L1CAM positivity was not significantly associated with disease free survival (HR 2.08; 95 % CI: 0.85–5.10, *p =* 0.11) (Table 4).

**Table 4:** Adjusted hazard ratios (HR) with 95 % confidence intervals (CI) of disease-free and overall survival (*n =* 388)

	Disease-free survival			Overall survival		
	HR	95 % CI	P-value	HR	95 %	*P*-value
L1CAM						
Negative						
Positive	2.08	0.85–5.10	0.11	1.81	0.79–4.11	0.16
FIGO stage						
Ia						
Ib	1.38	0.68–2.80	0.37	1.62	0.87–3.03	0.13
Age						
< 60 years						
> 60 years	1.85	0.75–4.54	0.18	4.15	1.46–11.7	0.01

In the subgroup that did not receive adjuvant chemotherapy, L1CAM positivity was significantly associated with disease free survival in univariate analysis (HR 3.2, CI: 1.24–8.56, *p =* 0.02). This significance was maintained when we controlled for age and stage (HR 2.9; 95 % CI: 1.08–7.56; *p =* 0.04). In patients who had received adjuvant chemotherapy, there was no such association (HR 0.68; 95 % CI: 0.07–6.76, *p =* 0.74).

In total, 41 patients died during follow-up. There were seven deaths (20.0 %) in the L1CAM positive group, and 34 deaths (9.6 %) in the negative group (*p =* 0.057) (Table 2). Median overall survival was not reached (data not shown). For the total group, L1CAM expression was not significantly associated with risk of death. FIGO stage and age were the only prognostic factors significantly associated with risk of death in univariate analysis (data not shown). The association with L1CAM expression remained non-significant, also in multivariate analysis (HR 1.81; 95 % CI: 0.79–4.11, *p =* 0.16) (Table 4).

## Discussion

This study aimed at the validation of L1CAM as marker of poor disease-free and overall survival in early stage endometrial cancer. The overall recurrence rate was low with only 9 % recurrences in the cohort as a whole and 17 % in L1CAM positive tumors. In the total cohort, neither disease-free nor overall survival differed significantly between L1CAM positive and negative patients when controlled for other prognostic factors. In a subgroup analysis of patients who had not received adjuvant chemotherapy, we found L1CAM expression significantly associated with disease free survival.

Several potential biases of retrospective studies were avoided as we a priori defined inclusion and exclusion criteria for the study sample. We also used the same immunohistochemical methods and a validated cut-off of 10 % for L1CAM positivity as described in the first report [3]. All cases underwent pathological review and were confirmed endometrioid adenocarcinomas. Surgical and adjuvant treatment were however defined by institutional guidelines and differed considerably between L1CAM positive and negative tumors. The differential impact of L1CAM expression in low, intermediate, and high-risk groups could not be analyzed in our study due to limited power.

The prevalence of L1CAM positive tumors in our study is lower than reported initially, but in line with the more recent reports from PORTEC and ENITEC [2, 6]. Our study confirmed that L1CAM expression of the tumor is associated with poor differentiation and patients with L1CAM positive tumors were more likely to belong to groups of higher risk of relapse. The association between L1CAM expression and higher grade has also been shown by two recent reports including endometrial cancer of various histologies and stages [7, 8]. In our study, differences in risk profile between L1CAM positive and negative patients led to considerable variations in the surgical and adjuvant treatment between the two groups. Also, as treatment was determined by institutional guidelines, treatment differed to some extent between the studies published to date. This is not unexpected as there is no overall agreement on the treatment of early stage endometrial cancer, but this makes it challenging to compare the reported outcomes. The large variations with regard to the practice of lymphadenectomy and the type of adjuvant treatment given are of particular importance here. At the Oslo University Hospital, pelvic and para-aortic lymphadenectomy is considered to be standard treatment for patients belonging to the intermediate and high-risk group. Consequently, 48 % of all women in our cohort had undergone at least pelvic lymphadenectomy. In the pooled analysis of PORTEC, crude rates of lymphadenectomy in the cohort as a whole and by L1CAM status have not been reported. However, systematic lymphadenectomy was conducted at the surgeon’s discretion in the PORTEC studies, and we may therefore assume that rates were lower than those reported here and by Zeimet et al. The ENITEC study has so far not reported on the primary surgical and adjuvant treatment and relative risks of relapse and death were given for all tumors in the study, including type II and more advanced stages of endometrial cancer. Even though it is still uncertain whether lymphadenectomy in itself confers a benefit in survival in early stage endometrial cancer, it may certainly lead to the upstaging of some patients. Our population of L1CAM positive tumors may therefore contain a higher proportion of true stage I patients while the study populations of both the other studies may have contained more patients with undetected lymph node metastasis who generally confer a higher risk of relapse, especially distant relapse. The fact that only 2 % of the patients in our cohort presented with distant relapse compared to 8 % in Zeimet et al. confirms this hypothesis. Another factor explaining the lower HR for disease free survival found in our study may be the use of adjuvant chemotherapy to high-risk patients in our study.

High-risk early stage endometrial cancers were mainly treated with adjuvant radiation therapy (VBT +/- EBRT) in both published studies to date. Only 3 % of the L1CAM positive patients and 2 % of the L1CAM negative patients had received adjuvant chemotherapy in the Austrian cohort [3] compared to 34 % and 7 % in our cohort, respectively. In the PORTEC studies, adjuvant chemotherapy was not given at all. Chemotherapy has been shown to decrease the risk of distant relapse compared to radiotherapy and there is also evidence for better disease specific survival after chemotherapy when added to radiation [9, 10]. In the group of patients not receiving adjuvant chemotherapy, we found a statistically significant HR of 2.9. This is in agreement with the previous studies showing that positive staining for L1CAM is related to an increased risk of relapse. However, these results have to be interpreted with caution as the number of relapses in the population as a whole was low compared to the other studies. The routine administration of adjuvant chemotherapy to patients in the high-risk group may have diluted the prognostic effect of L1CAM staining for the total group of patients in our study. The absolute differences in relapse between L1CAM positive and negative patients were small independent of the treatment with adjuvant chemotherapy or not, and the proportion of patients with distant relapse in L1CAM positive tumors when not given adjuvant chemotherapy was similar to the cohort as a whole. The clinical impact of the significant association between L1CAM expression and relapse free survival for patients who were not treated with chemotherapy warrants further investigation. Prospective studies may be needed to elucidate the value of L1CAM in predicting distant metastasis and to further investigate the effect of chemotherapy in patients with L1CAM positive tumors.

In our study, consisting mainly of patients belonging to the low and intermediate risk group, where patients in the intermediate group routinely underwent lymph node staging, the absolute differences in clinical outcome between patients with L1CAM positive and negative tumors was smaller than initially assumed. This is important as the overall risk of relapse and disease-specific death in this population is low and the differences may not justify more aggressive treatment of patients with L1CAM tumors at this stage. Systemic adjuvant treatment of all patients with L1CAM positive tumors would be a substantial overtreatment. It is further uncertain, whether L1CAM status can reliably be evaluated pre-operatively as only the pre-operative assessment of a prognostic maker would allow tailoring of surgical treatment.

## Conclusions

Our report confirms that L1CAM is associated with a more aggressive tumortype and more distant relapses. However, in this cohort of predominantly low risk early stage endometrioid cancer, the overall risk of relapse and disease-specific death is low and patients at higher risk were treated accordingly based on histopathology. Therefore, L1CAM staining did not seem to be a clinically relevant marker of poor prognosis and indicator for further treatment in this study.

## Abbreviations

CI, confidence interval; EBRT, external beam radiation therapy; EC, endometrial cancer; ENITEC, European network for individualized treatment of endometrial cancer; FIGO, Fédération Internationale de Gynécologie et d’Obstétrique; HR, hazard ratio; L1CAM, L1 cell adhesion molecule; LVSI, lymphovascular space invasion; VBT vaginal brachytherapy
